# The Dysarthric Expressed Emotional Database (DEED): An audio-visual database in British English

**DOI:** 10.1371/journal.pone.0287971

**Published:** 2023-08-07

**Authors:** Lubna Alhinti, Stuart Cunningham, Heidi Christensen

**Affiliations:** 1 Department of Computer Science, University of Sheffield, Sheffield, United Kingdom; 2 Health Sciences School, University of Sheffield, Sheffield, United Kingdom; 3 Centre for Assistive Technology and Connected Healthcare (CATCH), Sheffield, United Kingdom; University of Auckland, NEW ZEALAND

## Abstract

The Dysarthric Expressed Emotional Database (DEED) is a novel, parallel multimodal (audio-visual) database of dysarthric and typical emotional speech in British English which is a first of its kind. It is an induced (elicited) emotional database that includes speech recorded in the six basic emotions: “happiness”, “sadness”, “anger”, “surprise”, “fear”, and “disgust”. A “neutral” state has also been recorded as a baseline condition. The *dysarthric* speech part includes recordings from 4 speakers: one female speaker with dysarthria due to cerebral palsy and 3 speakers with dysarthria due to Parkinson’s disease (2 female and 1 male). The *typical* speech part includes recordings from 21 typical speakers (9 female and 12 male). This paper describes the collection of the database, covering its design, development, technical information related to the data capture, and description of the data files and presents the validation methodology. The database was validated subjectively (human performance) and objectively (automatic recognition). The achieved results demonstrated that this database will be a valuable resource for understanding emotion communication by people with dysarthria and useful in the research field of dysarthric emotion classification. The database is freely available for research purposes under a Creative Commons licence at: https://sites.google.com/sheffield.ac.uk/deed

## 1. Introduction

One of the fundamental factors affecting the quality of people’s lives is their ability to communicate effectively. Interpersonal communication is important for people to be able to socialise, express their feelings and needs, ask questions, share thoughts, etc. Communication plays a vital role in defining who we are. There are different ways of communication and spoken language is one of the most common and significant. However, not everybody can communicate well using natural speech [1]. For example, people with speech disorders may lose their ability to produce intelligible or audible speech sounds. As a result, people with speech disorders may also suffer from low self-esteem and may be hindered from achieving their goals in education, employment, and life in general [1–3].

There are many types of speech disorders. The most common acquired speech disorder is dysarthria [3, 4]. It is defined as a neurological disorder that affects different aspects of speech production caused by weakness in the muscles responsible for speaking, miscoordination or inaccuracy of articulatory movements, or irregularity in the tone, steadiness, or speed. Dysarthric speech has been characterised prosodically as having monoloudness, monopitch, impaired ranges of fundamental frequency (F0), vocal intensity, and rate [4]. These prosodic impairments can significantly affect the intelligibility of the speech [5, 6].

Communication between people can be viewed simply, as the process of producing and receiving messages. Messages are formulated using different signs and codes that are in turn interpreted by the receiver [7]. To avoid any confusion of what is meant by verbal and nonverbal communication in the context of this paper, from here after, verbal communication is referred to the spoken words while nonverbal communication is referred to how these words are spoken (voice characteristics). The ability to communicate effectively relies on a number of aspects that are not only limited to the intelligibility of the spoken words. In particular, nonverbal cues play a critical role in the correct comprehension of the delivered message. For example, a sentence may have different meanings when it is spoken with different voice tones [8]. Thus, relying on communication with missing or ambiguous nonverbal cues may lead to problems as people cannot express all their feelings using words alone. Nonverbal information conveys part of a person’s feelings and emotions. Combining verbal and nonverbal communication results in a better perception of a speaker’s feelings and emotions [9].

With the emergence of augmentative and alternative communication (AAC) technology, people with communication disabilities have been given a way to support their communication. One of the recent advances in the AAC technology is the use of voice-driven AAC. The voice-input-voice-output communication aid (VIVOCA) which is a communication aid that recognises disordered speech and reproduces it in a synthesised voice is a form of voice driven communication aid. It helps not only in retaining the responsiveness, speed, and naturalness of the speech communication, but also opens new doors into recognising nonverbal information [10–12]. Adding expressiveness to the synthesised voice will enhance the users’ communication experience and social relationship. Many people with dysarthria show a strong preference for using their own voice (also known as *residual voice*) when they communicate as it is the natural means of communication [13].

Communicating emotion is essential in building and maintaining relationships. The ability to recognise emotion is an important component in social interaction. People can express their emotions through a number of different modalities including speech, voice characteristics, facial expressions, and gestures. Automatic speech emotion recognition (SER) gained a lot of interest due to its increasingly important role in many fields including assistive technology and healthcare. There is a huge body of work done by researchers to automatically recognise emotions from human *typical* speech including recognising discrete emotions, recognising positive and negative emotions, and recognising nonverbal sounds such as cries and laughter [14–18].

Emotions have been investigated in dysarthric speech. The authors in [19] found that listeners faced a lot of difficulties recognising most of the emotions produced by English speakers with dysarthria caused by Parkinson’s disease (PD) in comparison to typical speakers. Although [20] found no significant difference in recognising emotions by listeners between two groups of Dutch speakers where emotion identification were above random for both groups, it was shown that it was a difficult task regardless of the type of speech. On the other hand, a number of studies investigated the prosodic and phonatory features of dysarthric vocalisation. Despite having speech which is less intelligible, many studies show that even with the limited phonological and prosodic dimensions, many people with dysarthria have enough control to signal prosodic contrast on different tasks. For example, several studies investigated the ability of people with dysarthria caused by either cerebral palsy (CP) or PD to signal question-statement contrast in different languages [19–25]. Other studies investigated the prosodic and acoustic characteristics such as the fundamental frequency (F0), intensity, and speech rate of speakers with dysarthria in comparison to typical speakers [26–33]. The ability to perform boundary marking by people with dysarthria caused by PD and contrastive stress by people with dysarthria caused by CP and PD was examined [19, 20, 34]. In addition, the ability of people with dysarthria caused by PD to produce a perceptually detectable accent was also investigated and their strategy was compared to typical speakers [35]. In addition, the authors of this paper have investigated the ability of people with dysarthria due to CP and PD to communicate emotions in their speech [36] and the ability to automatically recognise these emotions [37] using the database described in this paper. Although speakers with dysarthria can differ from speakers with typical speech in their prosodic characteristics, most studies showed that they were able to perform different productive prosodic skills similar to healthy control speakers. High variability among speakers was observed in some of these studies such as their ability to vocally mark questions. These studies show that they may have enough control to convey emotions and show intentions in their speech which opens up new horizons for improving communication aids.

Whereas there exist a few databases on dysarthric speech such as the TORGO Database [38], the Nemours database [39], the homeService corpus [40], and the Dutch dysarthric speech database [41], they are not emotional databases and therefore cannot serve the purpose of analysing emotions in dysarthric speech nor developing automatic emotion classification systems. Thus, a proper database, called the Dysarthric Expressed Emotion Database (DEED), a parallel multimodal (audio-visual) database of dysarthric and typical emotional speech in British English was developed. As has been explained previously, emotions can be expressed using different modalities including speech and facial expressions. The inclusion of the visual part in DEED will allow the investigation of the ability of people with dysarthria to communicate emotions through facial expressions in addition to speech. Although this paper will be presenting both modalities, the focus of the current paper is on evaluating just the audio part while the video evaluation is planned for a subsequent paper.

The fact that emotional states are caused by many factors is the reason behind the difficulty of collecting samples of people under a particular emotional state [42]. Over the past decades, there has been considerable debate over the type of methodology that should be used. The studies on vocal emotional expressions fall into one of the following three main paradigms: natural vocal expressions, induced (elicited) vocal expressions, and acted (simulated) vocal expressions.

### 1.1 Natural vocal expressions databases

Natural vocal expressions are those emotional expressions that were recorded during different states of naturally emotional situations. An example of these kinds of databases includes those who were recorded for pilots in dangerous flight situations. Another example is recordings from different shows on TV and news where journalists report events that elicit emotions. Although this could be seen as the ideal research paradigm because of its high ecological validity, there are a number of methodology issues that are related to these kinds of recordings:

- These recordings are usually very brief and are taken from single or limited number of speakers.
- Mostly, suffer from poor recording quality.
- The task of determining the underlying emotion can be challenging.
- Mostly, protected by copyright laws and privacy policies which prevent them from being shared and distributed widely.
- The task of processing the data is very challenging due to the lack of control on the different aspects that are related to the recording settings such as: the environment and the background noise where the recorded data is taking place, the position of the microphones and cameras, the content, and the conveyed emotion [43, 44].

Examples of corpora that fall under this category are the Belfast natural database [45], the Geneva airport lost luggage study [46], and the databases in [47] and in [48].

### 1.2 Induced (elicited) vocal expressions databases

Eliciting emotional states in a speaker and recording his/her speech is another way followed by some researchers to study the effect of emotions on the voice. This intermediate paradigm falls between the natural and acted paradigms. A number of techniques have been used to induce specific emotions. For instance, [49–52] ask the speakers to recall previous events and experiences where they have felt certain emotional states known as self-induction, presenting emotional materials such as pictures, films, and stories that trigger emotions [53–55], ask the speakers to perform selected emotional scripts or improvised hypothetical scenarios that are designed to trigger certain emotions [43], and solving specific problems under different induced levels of stress and/or time pressure [56, 57]. One of the advantages of this methodology that makes it favoured by experimental psychologists is the degree of control it offers which leads to having more consistent speech samples. However, this methodology contains a number of drawbacks, including relatively weak effects are often produced from following these procedures and following the same procedure on different people does not necessarily mean that the same emotional effect will be produced by all of them [44].

Since this paradigm needs to be aided by additional resources, it cannot be seen as that much different from the acted paradigm [42].

### 1.3 Acted (simulated) vocal expressions databases

This strategy is the most preferred strategy in the field of collecting emotional speech databases [44]. Speakers who are sometimes professional actors, lay actors, or non actors are asked to produce emotional verbal expressions that are usually based on standard verbal content [58–66]. There have been some raised doubts about this methodology that falls mainly under two points. First, this methodology may produce more intense or exaggerated emotional expressions when compared to the ones that result from induced and natural methodologies. Second, actors often tend to overemphasize powerful and obvious cues while they miss more subtle cues that help in differentiating discrete emotions in natural expressions [58]. It could be argued, however, that with the existence of social constraints over emotional expressions and unconscious tendencies toward self-presentation, all the public expressions could be seen to some extent as "portrayals" [44]. In addition, since the listeners recognise reliably the emotional states from the acted speech, a reflection of part of the normal expressions at least could be assumed [44]. This strategy is still being followed and used by researchers in the field and the main advantage of this strategy is the full control that it provides which result in:

- Having high quality recordings that enable later speech processing and analysis.
- Having utterances with unambiguous emotional states.
- Allowing comparisons to be made among emotions and speakers as the same utterances have been recorded by all speakers.
- The ability to recruit a reasonable number of speakers over targeted group to act all kinds of emotions that are under the study to enable generalisation [67].

As can be seen each paradigm has its advantages and disadvantages and what really can be seen as the determinant factor is the goal of the research. Naturalness may not be the optimal methodology of some research goals. In addition, more attention should be paid to the strategies followed when selecting the acted and elicited methodologies to insure that the results are adequate reflection of reality. As part of these strategies, a natural database should be used as a comparison and a way to help the development of acted and elicited databases (Douglas-Cowie et al., 2003). Also, since studying emotion is a multidisciplinary area with many variables to consider, a single corpus may not be sufficient to address all the open questions but a set of databases that comply with the core requirements and standards would probably do [43].

If we look again into these three methodologies, then it would be clear that on one hand we have the natural databases. On the other hand, we have the acted databases. It is unclear, however, where would the elicited speech databases stand. Would it be something in between, more towards the acted speech databases, or more towards the natural speech databases? Would the technique used in the elicitation process affect this categorisation? This uncertainty is probably a result of the lack of clear definition of what is considered as an elicitation technique and what is not. For example, employing the self-induction technique by asking the speakers to remember a situation from the past where he/she felt a specific emotion and give the speaker time to put him/herself into that specific emotion before the start of the recordings, would presumably be very similar to what some speakers implicitly do in the acted approach. Speakers in the acted approach cannot just start recording emotional speech directly, there must be some kind of internal eliciting approach they follow that helps them produce the required emotional state even if they have not been explicitly directed to a specific approach. This leads to the following two questions being raised: is there purely acted speech and are the acted and elicited approaches two distinct approaches or are they somehow part of each other?

This grey area may lead to a confusion in the databases’ categorization process. The Berlin database of emotional speech [59] for example, has been categorised as an acted emotional speech database, even though self-induction approach was adopted as their database recording methodology. While at the same time, an emotional speech corpus in Hebrew [52], and the Ryerson Audio-Visual Database of Emotional Speech and Song (RAVDESS) [51], has been categorised as semi-natural (induced) speech emotional database, although, a self-induction approach was adopted in the database recording. Another example, is the SAVEE database that has been categorised as acted emotional speech although the emotion stimuli, including photos and short video clips, have been presented to the speakers before recording the emotional utterances [62]. This methodology could be seen by many as induced methodology rather than acted.

There is a need for researchers in the emotion and psychology field to come up with a clear definition and set boundaries that helps in distinguishing between these two approaches.

One of the very important questions is which methodology should be adopted to develop DEED to ensure that it is reliable and valid? Since there are no available emotional databases on dysarthric speech, there are no means of practical comparisons on the methodologies that are best to follow. Natural methodologies will not be appropriate in forming this database due to the many problems related to natural database recordings discussed above. More layers of difficulties are added to these problems given the fact that the recordings here are of atypical speech. For example, the task of determining the underlying emotion would be much more challenging for dysarthric speech than it would be for a typical speech—in fact, this point is the key research question guiding this work. Since this database is the first of its kind, having such an ambiguity in determining the expressed emotions would not help in constructing a reliable database. In fact, having a natural database of dysarthric emotional speech may make the distribution of the database to the research community difficult or impossible due to ethical and privacy issues. Therefore, the choice is between using the acted or the elicited methodology. According to the discussion made above, two main points have been highlighted:

- Although many emotional databases have been categorised as acted, when looking at the way they were recorded, it is clear that some form of elicited methodology has been used, despite this often being miscategorised as acted.
- Is there really a pure acted methodology? Even if no explicit elicitation techniques have been used such as presenting emotional stimuli, it cannot be guaranteed that at the very least self-induction techniques have not been used by the speakers even if they have not been asked to do so explicitly. In fact, one could argue that without self-induction, the acting would be quite poor.

As a result, one can see that the acted and elicited methodologies are very closely related and in practice they can rarely be thought of as two distinct methodologies.

Therefore, based on the nature of the speech disorder (dysarthria), a strong argument in favour of the elicited methodology can be made. The main issue that is in disfavour of the acted and some elicited speech methodologies, is the concern of producing more intense or exaggerated emotional expressions when compared to the natural methodology. However, in the case of disordered speech in general and dysarthric speech in particular, this is not considered an issue. This is because people with a speech disorder tend to exaggerate the way they speak anyway in an attempt to try to get their messages across. Therefore, the gap between the natural and elicited way is not that big. With the power of control offered by the elicited methodology, adopting the elicited approach in the development of dysarthric emotional speech database looks highly valid and thus it was selected as the methodology for recording DEED.

DEED design enables the:

- Analysis of the features used by people with dysarthria when communicating different emotions [36].
- Comparison of these features with that used by people with typical speech when communicating the same emotions [36].
- Development of automatic emotion classification model for dysarthric speech [37].
- Investigation of the ability of people with dysarthria to communicate emotions through facial expressions.
- Development of dysarthric speech recognition systems.
- Application of the findings in the development of emotional speech synthesis for the dysarthric speech in a voice-input-voice-output communication aids (VIVOCAs) [10, 11].

To enable further research, this paper introduces and describes the creation of DEED and reports its validity based on the results of the performed subjective and objective evaluations. Although the main purpose of this database is to enable the investigation of automatically classifying emotions in dysarthric speech, it is important before that, to evaluate the DEED recordings subjectively. Obtaining human performance on the collected database is an approach that is followed in emotional typical speech. This will help in determining the task difficulty level for humans. It will also provide a benchmark for automatic emotion recognition models.

The remainder of this paper is organised as follows. Section 2 describes the design and development of DEED. Section 3 presents the validation of DEED stimuli including all the details of the performed subjective and objective evaluations. Finally, Section 4 includes the conclusion.

## 2. Corpus design

DEED is an audio-visual British English database of emotional speech that contains both typical and dysarthric speech. A controlled approach has been adopted for the design and development of this database. The below subsections will discuss the adopted approach. The recording approach of DEED has been ethically approved by the University of Sheffield, UK. Before any recording session, a written consent form has been obtained from every participant. In addition, the individual in Fig 3 in this manuscript, discussed later, has given written informed consent (as outlined in PLOS consent form) to publish his photo.

### 2.1 Scope

**Fig 1:**
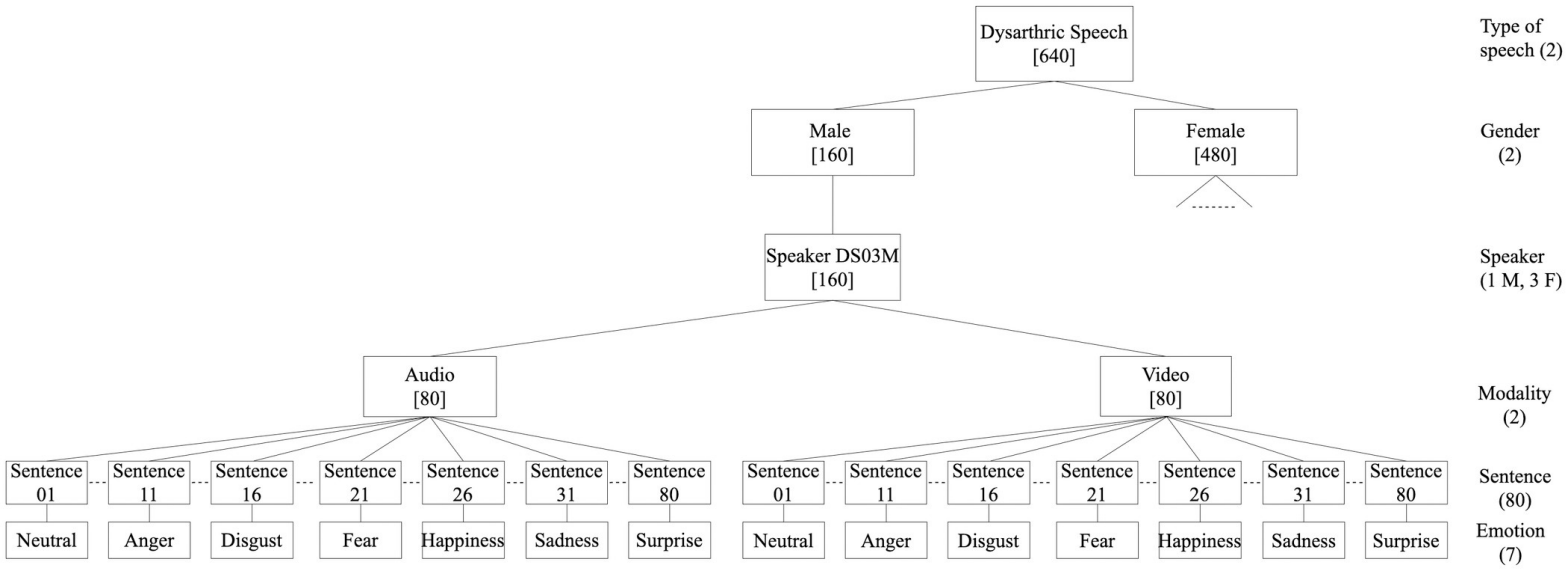
Tree diagram of the design of the DEED typical speech corpus part. (The numbers written between square brackets indicate the total number of files for that level).

**Fig 2:**
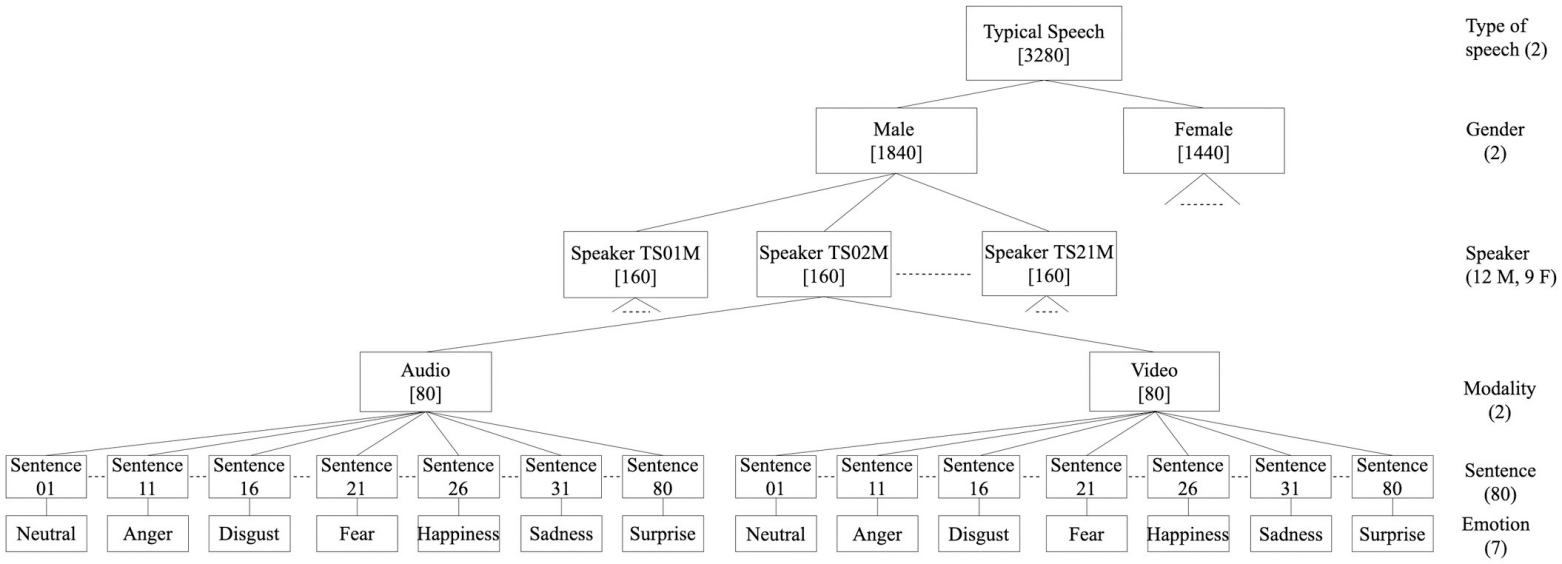
Tree diagram of the design of the DEED dysarthric speech corpus part. (The numbers written between square brackets indicate the total number of files for that level).

To the best of our knowledge, DEED is the first database of its kind. It is not only that it contains multimodal (audio and video) emotional dysarthric speech but also that it contains emotional typical speech. Both kinds of speech were recorded in the same recording studio using the same settings. This allows a fair comparison and analysis to be made between the two types of speech. Table 1 presents the details of DEED audio recordings including the details of the typical speech part and the dysarthric speech part for the four speakers with dysarthria: DS01F, DS02F, DS04F, and DS04M. The table includes the details of the number of audio recordings (utterances), number of speakers, the total length of the recordings, and the average length of utterances in seconds. Figs 1 and 2 show the tree diagram of the design of DEED typical speech and dysarthric speech corpus parts, respectively. As can be seen, it is a collection of recordings of people reading sentences in different emotions including anger, happiness, sadness, surprise, fear, disgust, and in neutral. The numbers written between square brackets indicate the total number of files for that level. All speakers have been audio and video recorded, except one of the male typical speakers who was only audio recorded. As can be seen, the size of *DEED-typical* speech part may make it suitable for different machine learning approaches. Although the main reason for developing this database is for dysarthric emotion classification purposes, it can be used for training ASR for dysarthric and typical speech.

**Table 1:** The details of DEED audio recordings.

**Typical speech**				
**Number of audio recordings (utterances)**	1680 (80 utterances per speaker)			
**Number of speakers**	21 speakers (12 male and 9 female)			
**Total length of recordings**	1.268 hours			
**Average length of utterances in seconds**	2.2			
**Dysarthric speech**				
**Speakers’ ID**	DS01F	DS02F	DS04F	DS03M
**Number of audio recordings (utterances)**	80	80	80	80
**Total length of recordings in minutes**	15.393	4.008	3.805	3.599
**Average length of utterances in seconds**	11.6	3.0	2.9	2.7

For speakers’ ID: DS = dysarthric, (01,02,03,04) = speaker id, F = female, and M = male).

### 2.2 Selection of speakers

There were three groups of participants in this study: speakers with dysarthria associated with cerebral palsy (CP), speakers with dysarthria associated with Parkinson’s disease (PD), and speakers with typical speech. All participants were recruited using advertising emails sent to special email lists, flyers, and word of mouth in the area of Sheffield, UK. The inclusion criteria for all of the three groups were that the participant must be a native British English speaker, over the age of 18, and have no known cognitive problems and no known literacy difficulties. None of the participants were professional actors. Informed consent was obtained from all participants.

#### 2.2.1 Speakers with dysarthric speech

Two groups of participants with dysarthria were included in this study. The first group contained 1 female speaker with severe dysarthria associated with CP. The second group contained 2 female speakers and 1 male speaker with dysarthria associated with PD. Recordings of speakers with PD were taken while they were under the anti-Parkinsonian medications effect. Table 2 lists the details of the speakers and their dysarthria severity levels.

**Table 2:** Speakers’ description.

**Speakers with dysarthria**				
**Type of dysarthria**	**Speaker ID**	**Gender**	**Age**	**Dysarthria severity** */ time diagnosed
Spastic dysarthria (CP)	DS01F	Female	65 years	Severe / from birth
Hypokinetic dysarthria (PD)	DS02F	Female	71 years	Mild / 10 years
	DS04F	Female	68 years	Mild-to-moderate / 10 years
	DS03M	Male	66 years	Moderate / 9 years
**Speakers with typical speech**				
**Gender**	**Number of speakers**	**Age**		
		**Mean**	**SD**	**Range**
Female	9	34.00	13.26	20–56
Male	12	35.67	16.81	19–70

*The dysarthria severity levels indicated in the table are informal judgments by the authors.

#### 2.2.2 Speakers with typical speech

Twenty-one speakers with typical speech were included in this study, 12 male and 9 female. Table 2 lists the details of the speakers of this group. All twenty-one speakers have been audio and video recorded except one male speaker who was only audio recorded.

### 2.3 Selection of emotions

With respect to the set of emotions captured, the widely adopted approach is to capture a small set of ‘basic’ emotions [42]. Most of the discrete emotion models are taken from Darwin’s "The Expression of Emotion in Man and Animals" [68]. This discrete emotion pattern approach has been popularised by scholars in this field: Tomkins, Ekman and Izard [69–78]. The Geneva airport lost luggage study [46], the Danish Emotional Speech (DES) database [79], the Berlin database of emotional speech [59], the eNTERFACE’05 audio-visual emotion database [53], the SAVEE database [62], and the RAVDESS database [51], to name a few examples of databases that adopted this approach in the development of their databases. Two other approaches have emerged as a result of the debate on the range of emotions covered. The first approach is to cover a larger set of emotions and sometimes distinguish between different forms of an emotion. [58] is an example of a study that followed that approach where cold and hot anger have been differentiated. The second approach is to cover a narrower set of emotions and therefore study it in depth. The most prominent databases that follow this approach are stress oriented. [55, 56] are examples of studies that followed this approach where different levels of stress have been investigated.

The authors in [80] found in their review that the most common recorded emotions ordered in decreasing order are: anger, sadness, happiness, fear, disgust, surprise, boredom and joy. Also, despite the number of different emotions, [81] found in their review of sixty-four emotional speech databases, that the majority have limited their recordings to five or six emotions. It is important in this aspect to consider that emotional life is strongly influenced by culture and are subject to social rules, display rules, and feeling rules [82, 83]. Also, moderate emotional states are more expressed during daily communication rather than full blown basic emotions [42]. Therefore, if the developed technology is to be used for everyday life, it is important to consider the set of emotions that occurs frequently and most importantly to get across for people with dysarthria rather than studying emotions that occur rarely in everyday situations [44, 84].

All of the above approaches have their defenders. However, since the debate remains unsettled, the six basic or primary emotions: “happiness”, “sadness”, “anger”, “surprise”, “fear”, and “disgust” have been selected for the development of DEED. A “neutral” state has also been added as a baseline condition. Another reason for this selection of emotions is that these sets have been widely adopted in most existing databases [51, 53, 62, 85, 86].

### 2.4 Stimuli

The text material is a subset of the material used in the SAVEE database [62]. Some of the long sentences were excluded from the adopted set of sentences, as it might have been difficult for some people with dysarthria to be able to speak them all. “He ripped down the cellophane carefully, and laid three dogs on the tin foil.” is an example of one of the long sentences that was excluded. The text material consisted of 10 TIMIT sentences per emotion, giving a total of 60 TIMIT sentences, such that: 3 common that were the same for each emotion, 2 emotion-specific and 5 generic sentences that were different for each emotion. The 3 common and 2 x 6 = 12 emotion-specific sentences were recorded as neutral in addition to 2 neutral sentences and 3 generic sentences. This gives a total of 20 neutral sentences. Therefore, a total of 80 utterances per speaker is recorded. The SAVEE database has a total of 120 sentences per speaker [62]. A list of DEED sentences for anger, disgust, fear, happiness, sadness, surprise and neutral emotions can be found in S1 Table.

The sentences were divided into fourteen blocks, where each block contained 5 sentences from the same emotion, except for the neutral state where the block contained 10 sentences. Each recorded set began with a neutral block followed by one block from each emotion in the following order: anger, disgust, fear, happiness, sadness, surprise. This gave a total of two rounds. This division procedure was applied to help in avoiding bias caused by speakers’ fatigue and to ensure that speakers, mainly those who have dysarthria and could not record the whole set of sentences, would at least have been through recording one round which includes a subset of the sentences that covers the full set of emotions.

The stimuli presentation consisted of three main stages: task presentation, emotive video presentation, and sentence presentation. In the task presentation stage, the emotional state that the speaker should perform next is presented as a text on a screen in front of the speaker for around 2 seconds. The emotive video presentation stage consisted of playing a short emotive video clip to help the speaker elicit the target emotional state. Finally, in the sentence presentation stage, each sentence within the current block of sentences is presented on the screen individually.

### 2.5 Emotion elicitation approach

In order to elicit specific emotions in speakers, emotion stimuli has been chosen as the eliciting technique. Very short video clips of emotion stimuli are presented in order to elicit specific emotions. The video clips that have been used are adopted from those used when recording the SAVEE database [62].

These video clips were taken from popular movies and television series. In addition to that, speakers were told that they can use Stanislavski’s emotional memory techniques where they are instructed that they can remember the details of a situation with the same emotion if they think it will help them to put themselves in a particular emotional state [87]. This follows standard protocols for recording such databases [51, 59, 62]. Speakers were given all the time they needed to put themselves into a specific emotional state. They have also been told that they can repeat a sentence as many times as they want until they feel satisfied with their performance. Speakers have been explicitly instructed to provide genuine expressions of emotions as they would do in typical everyday scenarios. No instructions or guidance were given in how a particular emotion should be expressed.

### 2.6 Data capture

**Fig 3:**
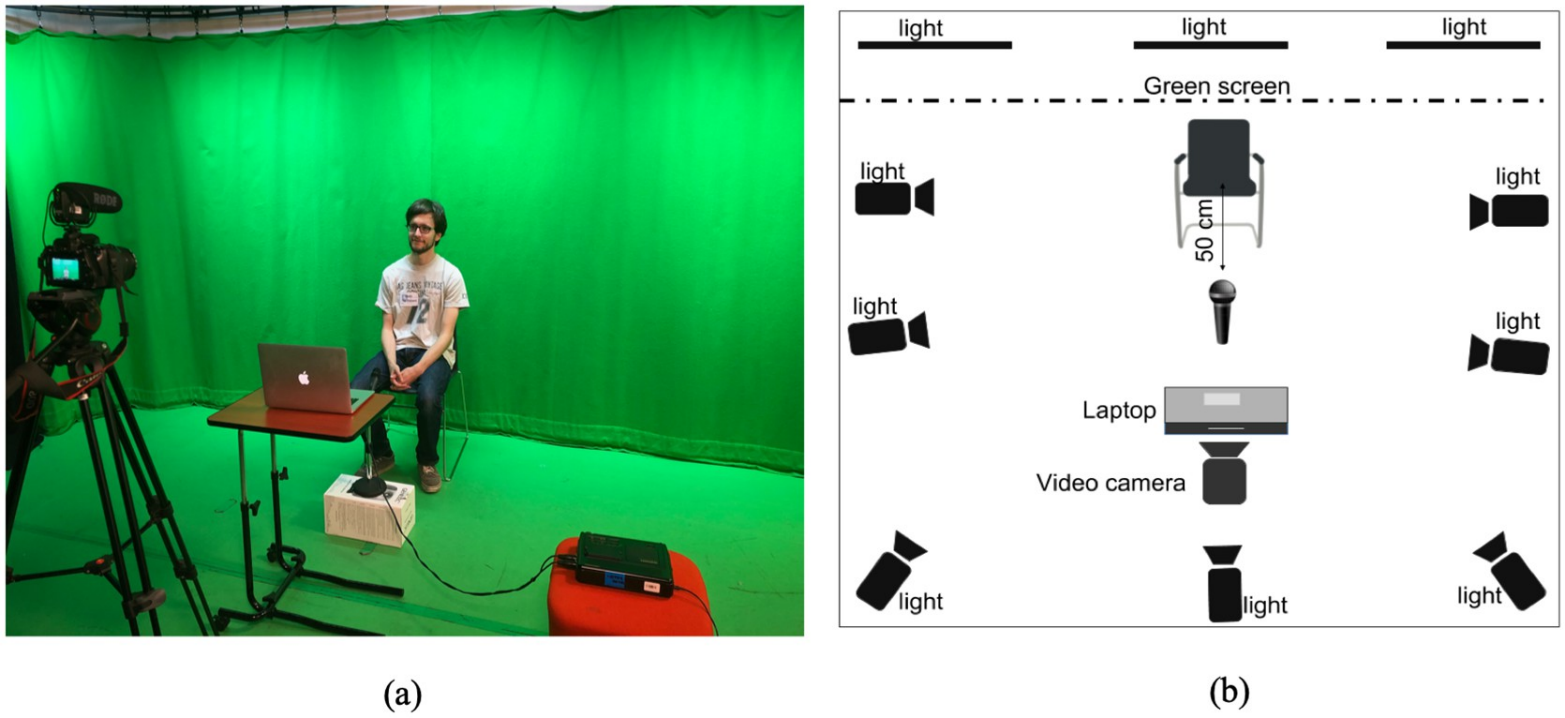
Recording studio physical setup.

The database recordings took place in a professional recording studio at the University of Sheffield over several months. Fig 3 shows the data capture physical setup. All speakers were sitting all the time during the recordings facing the camera. A green screen cloth has been used as the background. The microphone was placed 50 cm from the speaker. If needed, a break after finishing one round of the sentence blocks, although speakers were permitted to ask for a break anytime they felt they needed to. The duration of the break was entirely determined by the speaker. All speakers completed their recordings in one session.

### 2.7 Technical information

Speakers were recorded individually with a Canon EOS 80D DSLR Kit with 18-135mm IS STM Lens. Video files were saved in MOV format. The camera was placed around 1.5 metres from the speaker and zoomed as needed to only fit the speaker’s face, upper half of his/her body, and the green screen cloth. For the audio recording, a Marantz PMD 670 recorder was used. Audio files were saved in wave format. The microphone was placed approximately 50 cm from the speaker. Table 3 lists the camera and the recorder settings used in all the recording sessions. Speakers were illuminated using six ceiling mounted studio lighting rigs. The lightning levels were adjusted to provide full spectrum lighting and to reduce facial and body shadows. The prompting material was displayed on a 13 inch MacBook Air and placed on a Table 1 metre from the speaker. Each speaker audio file was exported from the recorder and sentence segmentation was performed manually using the Audacity software (http://audacity.sourceforge.net). Each speaker video file was exported from the camera and video segmentation was performed using FFmpeg multimedia framework (https://www.ffmpeg.org/ffmpeg.html).

**Table 3:** Camera and recorder settings.

**Camera Settings**	
ISO	500
Frame rate	25 fps
Shutter speed	25
Aperture	5.6
**Recorder Settings**	
Recording levels	4
Sampling rate	16000Hz
Audio channel	Mono

### 2.8 Description of the DEED files

DEED contains 1680 recordings from 12 male and 9 female speakers with typical speech and 320 recordings from 1 male and 3 female speakers with dysarthric speech. All speakers produced 80 spoken utterances.

#### 2.8.1 DEED filename convention

A unique filename has been given to each file in DEED. Each filename consists of 9 alphanumeric identifiers (e.g., An01TS02M). Each 2–4 part characters represents different information. Positions 1–2 represent the emotion. Each emotion has been identified using the first 2 characters as follows: Ne:Neutral, An:Anger, Di:Disgust, Fe:Fear, Ha:Happiness, Sa:Sadness, and Su:Surprise. Positions 3–4 represent the sentence number. DEED has 80 sentences, therefore, sentences are identified using numbers from 01 to 80. Positions 5–8 represent the speaker ID. Each speaker ID is composed of letters (TS or DS) and unique numbers (01, 02,..etc.,) to differentiate speakers. TS identifies a speaker with typical speech and DS identifies a speaker with dysarthric speech. Position 9 represents the gender of the speaker where M indicates a male speaker and F indicates a female speaker.

### 2.9 Download and accessibility

Since one of the aims of developing DEED is to provide the research community with a validated parallel database of typical and dysarthric emotional speech, it is available free of charge subject to non-commercial use under a Creative Commons licence at: (https://sites.google.com/sheffield.ac.uk/deed).

## 3. Validation of DEED stimuli

DEED was validated using subjective and objective evaluation. The following sections will provide the details of the methodologies and results of both evaluations.

### 3.1 Subjective evaluation of DEED

The subjective evaluation approach of DEED has been ethically approved by the University of Sheffield, UK. Before any experiment, a written consent form has been obtained from every participant.

#### 3.1.1 Participants

Twenty-two normal hearing participants who are native speakers of British English or have lived in the UK for at least 1 year were recruited. Table 4 presents the participants’ details. None of the participants were familiar with any of the speakers with dysarthria in DEED, except one participant who was somewhat familiar with speaker DS01F, where they have met a few times in the past.

**Table 4:** Characteristics of participants in evaluation.

Demographic	Value			
**Age**	**Mean**	**SD**	**Range**	
	32.69	12.06	18–59	
**Gender**	**Female**		14 (63.64%)	
	**Male**		8 (36.36%)	
**English proficiency**	**Native**	**Lived in the UK for more than 5 years**	**Lived in the UK for 3–5 years**	**Lived in the UK for 1–2 years**
	9 (40.91%)	3 (13.64%)	5 (22.73%)	5 (22.73%)
**Familiarity with dysarthric speech**	**Extremely familiar**	**Somewhat familiar**	**Slightly familiar**	**Not familiar at all**
	1 (4.55%)	5 (22.73%)	1 (4.55%)	15 (68.18%)

#### 3.1.2 Stimuli, apparatus, and procedure

The evaluated stimuli consisted of the audio part of DEED. The selected stimuli included all the recordings of the speakers with dysarthria in addition to the recordings of 8 typical speakers who were randomly selected from the DEED-typical speech part (five female and three male). More female typical speakers were chosen in the evaluation as DEED-dysarthric speech part contains more female speakers than male. The randomly chosen typical speakers were: TS09F, TS13F, TS16F, TS17F, TS20F, TS06M, TS111M, and TS18M. Therefore, the stimuli consisted of a total of 960 audio recordings of emotional speech. Although the main aim is to evaluate the dysarthric speech part of DEED, it was important to include recordings of typical speakers as a baseline assessment measure of the participants’ evaluation level and to see where this database stands in comparison to previously published emotional typical speech databases.

The evaluation process began with a face-to-face approach, where participants were invited to the University of Sheffield and seated in a quiet room in front of a 13-inch MacBook Air laptop. Participants listened to the stimuli using a pair of headphones. However, due to the Coronavirus disease (COVID-19) pandemic and the lockdown imposed to stop the spread of the virus, it was not possible to carry on the evaluation using the same approach. Therefore, an online approach was proposed and adopted. Given the task in hand, the selection of the platform to carry out the evaluation was very critical as the audio files need to be streamed with no distortion or modification. After comparing several platforms, the Zoom video conferencing platform was selected [88]. Zoom allows a lot of flexibility in the audio settings to fit different needs. To make sure that the audio is heard by the study participant without any distortion or modifications, the following advanced settings were set:

- Disable “automatically adjust audio volume”.
- Enable “original sound from microphone”: this will turn off audio enhancements such as echo cancellation and noise suppression, which is a very important feature for audio streaming.
- Disable “suppress persistence background noise”.
- Disable “suppress intermittent background noise”.

People who teach vocals and music rely on these advanced settings as well (https://www.thenakedvocalist.com/zoom-for-singing-teachers/, https://www.makingmusic.org.uk/resource/ zoom-online-rehearsals-vocal). In total, ten participants evaluated the data using the face-to- face approach, while the other twelve participated using the online approach.

Balanced evaluation sets were created using the following approach. All the 7 emotions were included in the evaluation. For speakers with dysarthria, each speaker’s recordings were divided into two equal sets in terms of the number of recordings resulting in having 40 recordings (utterances) per set. For the typical speakers, each speaker’s recordings were divided into five equal sets in terms of the number of recordings resulting in having 16 recordings (utterances) per set.

The evaluation was carried out at utterance level. Each participant was presented with a chosen set of 288 stimuli as follows: 1 set (40 utterances) from each speaker with dysarthria recordings and 1 set (16 utterances) from each typical speaker’s recordings except one participant who had evaluated the whole set of stimuli (960 utterances) and another participant who had evaluated the whole dysarthric stimuli (Set 1 + Set 2 for each speaker with dysarthria) in addition to one set from each typical speaker’s recordings. Each participant was presented with a set from each speaker separately. Within each set, there is a training set and an evaluation set. The purpose of the training set is to help the participant get used to the speaker style of speaking.

**Fig 4:**
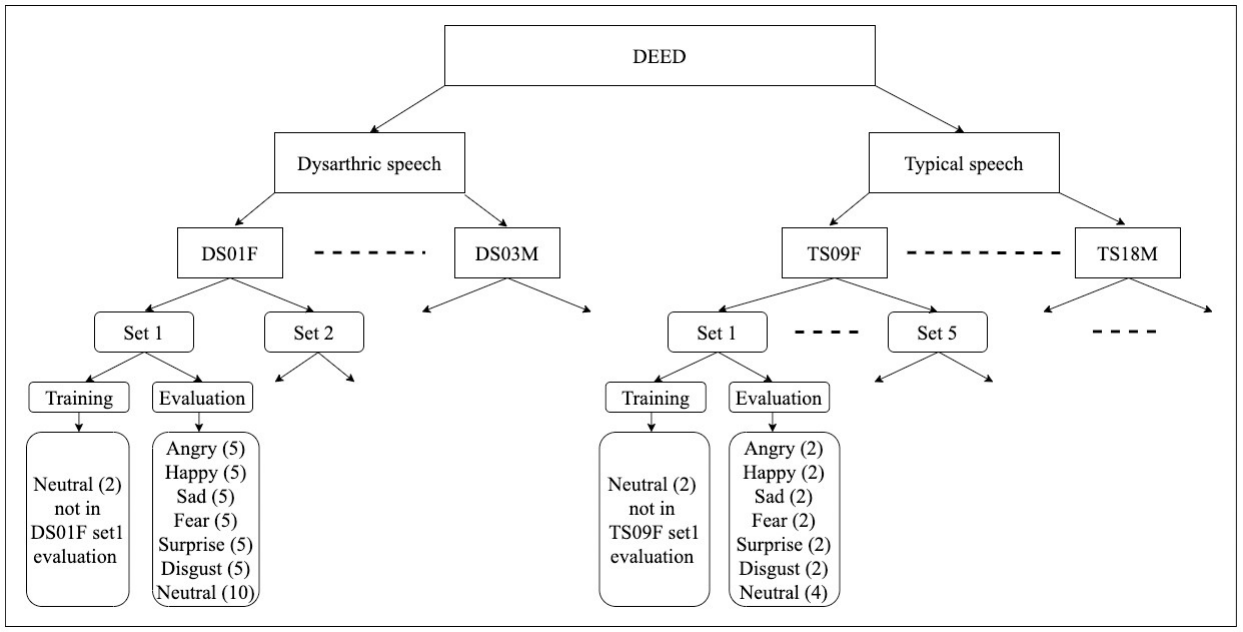
The division of DEED into sets for the purpose of evaluating the data subjectively. (The number between brackets indicates the number of utterances).

To remove the systematic bias from the responses of the participants, the order of utterance in each set was randomised such that each participant was presented with each speaker’s utterances in an order that is different from other participants. For example: participant 1 could be presented first with utterance 11 followed by utterance 45, and so on, from speaker DS01F while participant 2 was presented first with utterance 02 followed by utterance 72, and so on from the same speaker. Fig 4 illustrates the division of the data.

#### 3.1.3 Evaluation task

In addition to the verbal instructions, on-screen instructions were presented as well as follows: "1. Before you start the evaluation, you will be presented with 2 audio recordings of a person speaking in a neutral state, so you can get a sense of the speaker’s speaking style. 2. When the evaluation starts, you will be presented with audio recordings of a person speaking with different emotions. 3. After you listen to each recording, you will be asked to choose which emotion you felt was expressed. The instructions screen is shown in S1a Fig. As mentioned in the instructions, to help participants get used to the speaker’s style of speaking, participants were trained using two recordings from that speaker speaking in the neutral emotion before the evaluation of each set began. These two neutral recordings were not among the evaluation set. The training screen is shown in S1b Fig. Each recording was played only once. Participants were asked to choose an emotion using a forced-choice response format. The options were: angry, happy, sad, fear, disgust, surprise, and neutral. The options were disabled until the whole recording was played to ensure that participants listened to the whole recording before making a choice. It also helped in preventing participants from moving quickly through some stimuli or skipping some. The response evaluation screen before and after playing the recording is shown in S2a and S2b Fig, respectively.

#### 3.1.4 Results

Using the methodology described in the previous section, all the DEED-dysarthric speech recordings (320 utterances) were each evaluated 12 times, while the DEED-typical speech recordings included in the evaluation (640 utterances) were each evaluated 5 times. The average performance in the evaluation on speakers with dysarthria and typical speakers for the 7 emotions by all the participants are given in Tables 5 and 6 respectively.

**Table 5:** Average subjective evaluation performance (%) for 7 emotion classes on DEED-dysarthric speech.

Speakers	Accuracy (SD)	Recall	Precision	F-score
DS01F	22.81 (0.31)	20.83	20.48	19.93
DS02F	56.25 (1.46)	54.11	54.32	52.90
DS04F	40.00 (0.62)	35.65	35.28	33.88
DS03M	43.23 (2.60)	40.65	41.29	39.51

**Table 6:** Average subjective evaluation performance (%) for 7 emotion classes on subset of DEED-typical speech.

Speakers	Accuracy (SD)	Recall	Precision	F-score
TS09F	64.25 (3.32)	61.71	63.02	60.78
TS13F	55.75 (5.04)	54.57	56.58	53.43
TS16F	56.25 (6.66)	54.29	55.62	53.13
TS17F	51.25 (5.18)	48.00	52.04	47.09
TS20F	51.25 (4.81)	45.86	47.42	44.68
TS06M	55.00 (3.16)	50.00	51.81	48.68
TS11M	58.00 (4.23)	54.43	56.98	52.89
TS18M	56.12 (3.37)	53.97	55.72	52.69

The performance on typical speech is generally better than on dysarthric speech, as would be expected, on all speakers except on speaker DS02F, where the result is comparable to the results on some speakers with typical speech or even better than some of them. Nevertheless, the results on dysarthric speech are all above chance level (14%), which indicates the ability of listeners to perceive emotions communicated by speakers with dysarthria. Table 7 illustrates the average recall performance per emotion on all speakers with dysarthria. High accuracy was achieved for ‘anger’. A good accuracy was also achieved for ‘surprise’, ‘sad’, and ‘neutral’. While ‘happy’, ‘disgust’, and ‘fear’ are less accurate in being perceived.

**Table 7:** Average subjective evaluation recall (%) results per emotion on all speakers with dysarthria.

Emotion	DS01F	DS02F	DS04F	DS03M
**Anger**	22.50	93.33	70.00	70.00
**Surprise**	16.67	55.00	35.00	36.67
**Disgust**	7.50	29.17	20.83	25.83
**Fear**	14.17	35.83	11.67	21.67
**Happy**	14.17	41.67	10.83	27.50
**Sad**	34.17	52.50	30.83	41.67
**Neutral**	36.67	71.25	70.42	61.25

It is of interest to compare these results to the British English emotional database, SAVEE [62, 89]. SAVEE and DEED share a lot of similarities such as the language used for the recordings and the stimuli set. The main differences, apart from DEED being a parallel database of dysarthric and typical speech, are: that the number and gender of speakers as SAVEE has 4 male speakers only, that speakers in DEED are not actors while the speakers in SAVEE are actors, and the number of utterances in DEED is 80 per speaker which is a subset of the 120 utterances recorded per speaker in SAVEE. Table 8 presents the performance of the subjective evaluation over all four actors on SAVEE from [89], where each utterance was evaluated ten times (average accuracy = 66.45%) and the performance of the subjective evaluation over the randomly chosen eight typical speakers from DEED where each utterance was evaluated five times (average accuracy = 55.97%). Given the above stated differences, it is hard to directly compare the performance, however, it does give some insight into the quality level of DEED.

**Table 8:** Comparison of the subjective evaluation performance (%) on SAVEE and a subset of DEED-typical speech.

Database	Number of speakers	Average accuracy (SD)	Minimum accuracy	Maximum accuracy
SAVEE	4	66.45 (9.17)	53.20	73.70
DEED-Typical	8	55.97 (4.15)	51.00	64.25

**Fig 5:**
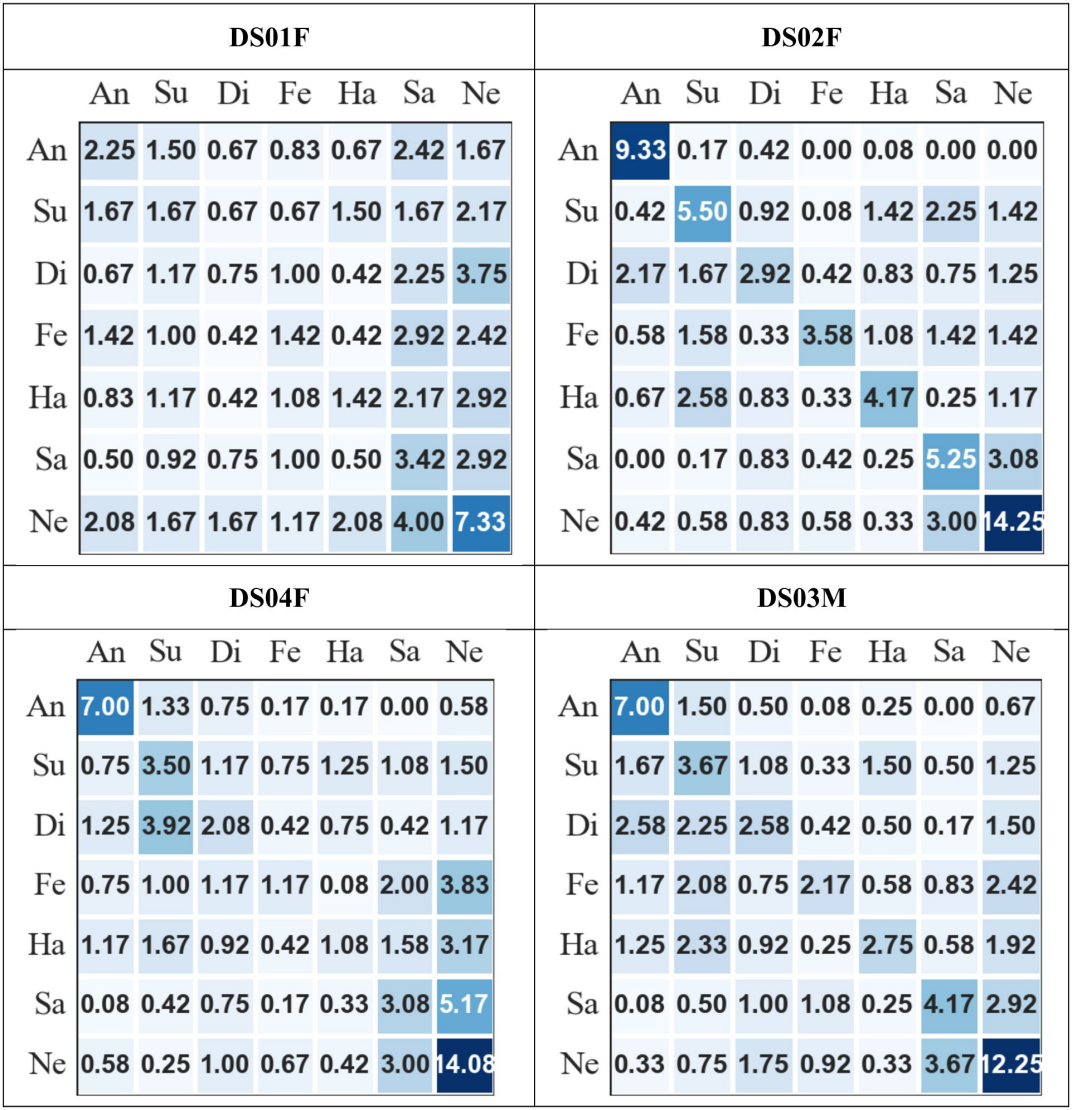
Average confusion matrices of the subjective evaluation for each speaker with dysarthria. (rows = actual emotions and columns = recognised emotions, An = angry, Su = surprise, Di = disgust, Fe = fear, Ha = happy, Sa = sad, and Ne = neutral).

**Fig 6:**
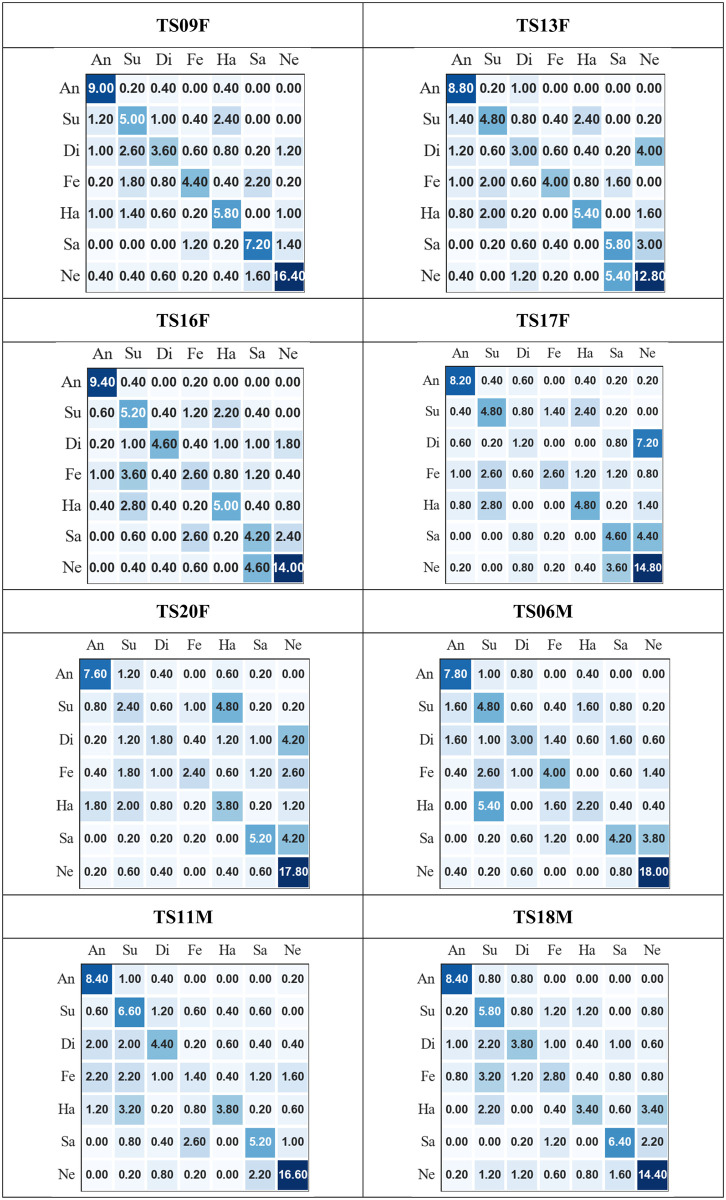
Average confusion matrices of the subjective evaluation for typical speakers. (rows = actual emotions and columns = recognised emotions, An = angry, Su = surprise, Di = disgust, Fe = fear, Ha = happy, Sa = sad, and Ne = neutral).

In addition to general performance metrics, confusion matrices are very important as they help in giving more insight to the recognition performance. Here, they can be used to highlight which emotions appear easier and harder to recognise and which emotions are more easily confused. Fig 5 presents the averaged confusion matrices on all speakers with dysarthria, where the rows present the actual emotions and the columns present the recognised emotions. From Fig 5, it is observed that, i) for all speakers, except for speaker DS01F, ‘anger’ is never confused with ‘sad’ and ‘sad’ is rarely confused with ‘anger’, ii) for all speakers, ‘sad’ and ‘neutral’ are most frequently confused with each other, and iii) for all speakers, except speaker DS01F, ‘anger’ appears to be the easiest emotion to recognise while ‘happy’, ‘disgust’, and ‘fear’ appear to be the most difficult emotions to recognise.. For speaker DS01F, it is observed that participants perceived most of the emotions as either ‘sad’ or ‘neutral’. The averaged confusion metrics for typical speakers are presented in Fig 6, where the rows present the actual emotion and the columns present the recognised emotions. It is observed that for all speakers, i) ‘anger’ is rarely confused with ‘sad’ and ‘sad’ is never confused with ‘anger’, ii) ‘sad’ and ‘neutral’ are most frequently confused with each other, iii) for all speakers, ‘anger’ appears to be the easiest emotion to recognise while ‘happy’, ‘disgust’, and ‘fear’ appear to be the most difficult emotions to recognise, and iv) ‘happy’ and ‘surprise’ are most frequently confused for each other.

#### 3.1.5 Discussion of the subjective evaluation

Although, the overall recognition performance on typical speech was generally better than on dysarthric speech, the performance on the latter was all above chance level (14%), even for speaker DS01F, who has severe dysarthria and low speech intelligibility. This was also the case when looking at the recall of each emotion for speakers with dysarthria presented in Table 7, except for ‘disgust’, ‘fear’, ‘happy’ for speaker DS01F and ‘fear’ and ‘happy’ for speaker DS04F, where the performance were at or below chance level. The highest recognition performance on dysarthric speech was for speaker DS02F, who has mild dysarthria.

Based on the confusion matrices on both types of speech, most of the patterns of confusion were similar. On both types of speech, ‘anger’ was found to be the easiest emotion to recognise, while ‘happy’, ‘disgust’, and ‘fear’ appear to be among the most difficult emotions to recognise. This was also observed from the recall per emotion presented in Table 7. The results of recognising ‘anger’ does not completely align with the findings by [19] who demonstrated the difficulty of English-speaking Canadians with dysarthria caused by PD to express emotions in their speech especially ‘anger’, ‘happy’, and ‘disgust’ were they were mostly perceived as ‘neutral’. It also does not tally with the findings from the survey conducted in [84] where ‘anger’ was chosen by almost half of the respondents, who were people with dysarthria caused by CP, as the most difficult emotion to communicate from their perspective. It is important, however, to highlight the fact that these evaluation results were obtained from audio data only and the survey asked about communication generally. It could be that some emotions have a higher *visual* component that make conveying them more easy or more difficult (depending on the disability) in a face-to-face communication situation. It was also found from the confusion matrices that ‘anger’ and ‘sad’ are not considered confusing pairs while ‘sad’ and ‘neutral’ are considered confusing pairs. This aligns with the findings in the literature from subjective evaluation of emotional data on typical speech [51, 90]. In general, the results of this evaluation show that listeners were able to perceive different emotions expressed by people with dysarthria and typical speakers which aligns to the findings by the authors in [20] who demonstrated the ability of speakers with dysarthria caused by PD to communicate emotions similar to typical speakers.

#### 3.2 Objective evaluation of DEED

**Fig 7:**
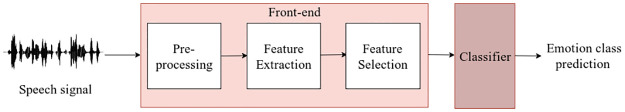
The general components of a speech emotion recognition system.

One of the objectives of recording DEED was to provide the community with a dataset that would enable research on the automatic recognition of emotions in dysarthric speech. The process of recognising emotions from speech is not a straightforward process for machines due to many factors including inter-speaker variability and the unavailability of an identified optimum set of acoustic features. A speech emotion recognition (SER) model can be viewed as a composition of a front-end and a classifier. Fig 7 depicts the general components of a SER model. The front-end consist of the feature extractor, this is also sometimes known as the parametrization process, and is responsible for obtaining different features from the speech signal that represent the data in a compact way by keeping the relevant information and discarding the irrelevant and misleading information which is a very challenging filtering process. The primary stages in the front-end subsystem are: signal pre-processing, feature extraction, and feature selection and dimension reduction [91–93]. The output of the front-end is then fed as the input to the classifier, which is usually based on a machine learning approach that is responsible for classifying the emotion expressed in the input. Classifiers get trained on a set of data and use what they have learned to classify new samples.

The aim of this objective evaluation is to explore the feasibility of automatically recognising emotions from dysarthric speech. It sets the baseline results for SER on the dysarthric speech part of DEED. Setting a baseline based on general techniques, previously used for typical speech, is important to be able to later on compare it to techniques turned specifically for dysarthric speech data. These baseline experiments also give an insight into the level of difficulty of the classification problem, and the performance of different classifiers using the same feature set on the dysarthric speech data. The below sections outline the baseline experiments and present all the details in terms of the database, the used feature set and classifiers. Finally, the results are presented and discussed.

#### 3.2.1 Data

**Fig 8:**
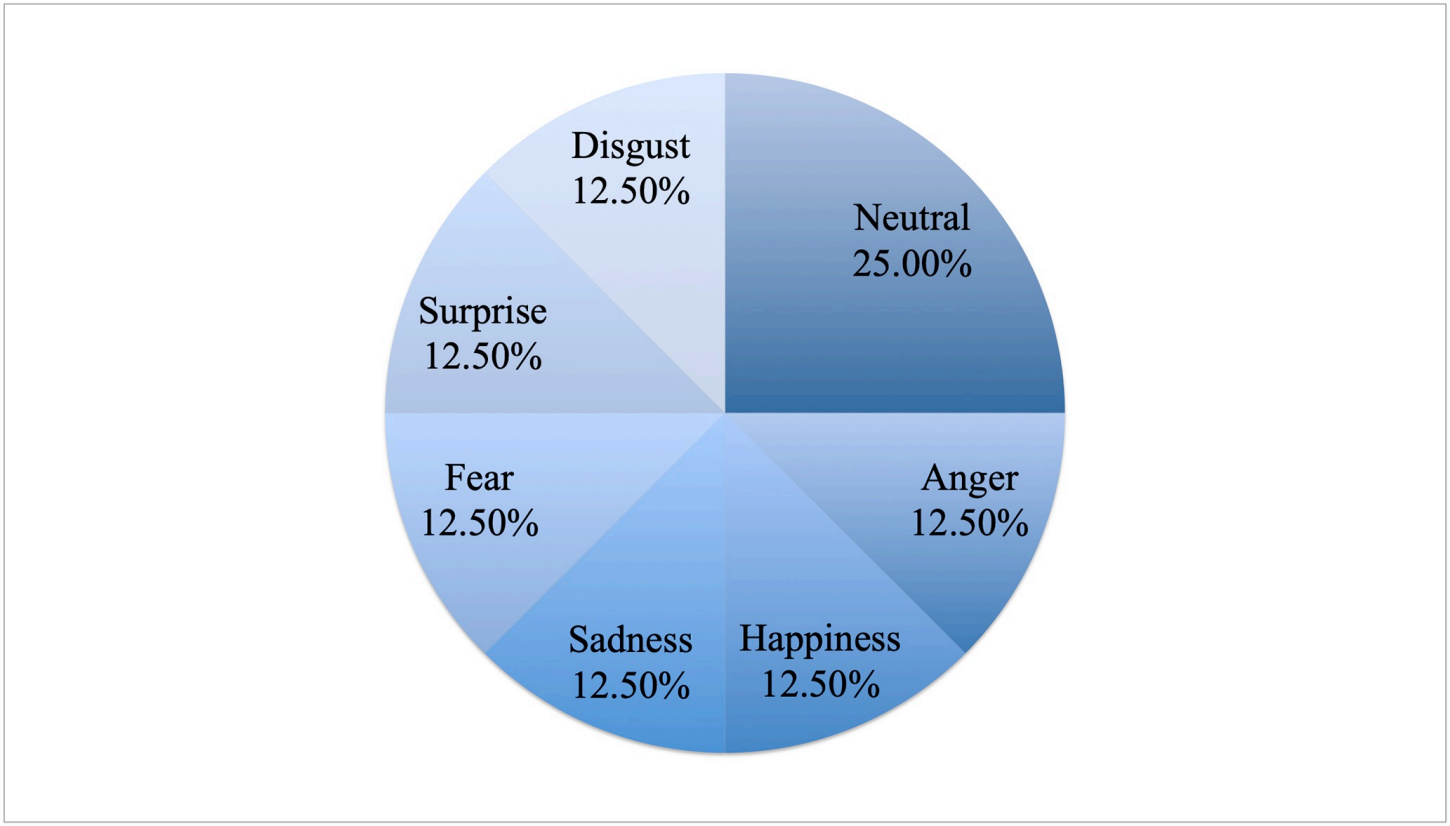
The distribution of the emotion classes in DEED.

These experiments were carried out on the dysarthric speech part of DEED. All the details about the database in terms of the speakers, emotions, and recording settings can be found in above sections. Fig 8 shows the distribution of the emotion classes in DEED. As can be seen, all classes have the same number of samples except for neutral where there are double the number of samples.

#### 3.2.2 Feature extraction

Due to the gap in the exact relation between physical acoustic features and perceived features, there is no agreement yet on the set of features that best describes emotions in speech. The level of comparability between results reported in the literature is low. Apart from the different classifiers, evaluation strategies, and databases used, the diversity in the sets of features is high. Even when two studies use the same features, one or more of the following is usually found: the underlying parameters used in the extraction are different, the exact parameters are not reported, the selection and implementation of global features (functionals) are not the same, and/or the strategies used in features reduction are different. The choice of the feature set contributes heavily to the performance of the SER model and it is one of its main challenges [94, 95]. Since these experiments aim to develop a baseline model for the dysarthric SER, the extended Geneva Minimalistic Acoustic Parameter Set (eGeMAPS) standard feature set was chosen as the feature set [96]. These features have been suggested to comprise the majority of the features that are related to emotions. The eGeMAPS has been widely used as a benchmark for emotion recognition studies [17, 97–103]. It contains spectral, prosodic, cepstral, and voice quality information such as F0, jitter, shimmer, harmonic differences, and MFCC for a total of 88 features. Also, using a standard feature set helps in making the results reproducible. The features were automatically extracted using the openSMILE toolkit [104] using the default parameters [96]. All features were standardised so that they have zero mean and unit variance.

#### 3.2.3 Classification

A discrete emotion classification approach was applied. 7-class and 4-class classification problems were reported. The 7- class classification problem included the full set of emotions while the 4-class classification problem included ‘angry’, ‘happy’, ‘sad’, and ‘neutral’ emotions only.

For classification, a speaker-dependent approach was used where the model is trained and tested using the target speaker’s speech characteristics. The results of each speaker are reported separately as have been done in other previous studies using different data sets [62, 89, 105, 106]. This helps in setting a clear baseline for each speaker. In this experimental study, the performance of the following classifiers were tested for the 7-class classification problem: One Versus Rest (OVR) SVM with RBF kernel and Logistic Regression (LR). For the 4-class classification problem, the performance of the following four classifiers were tested: (OVR) SVM with RBF kernel, LR, Decision Tree (DT), and Adaboost classifier. The selection of these traditional classifiers over deep learning models was based on the size of the data; as traditional classifiers usually require less data to work well while deep learning models are known to work better in the presence of sufficient training data. For SVM, the regularisation parameter (C) and the gamma coefficient of the kernel were set to 5 and 0.01, respectively. For LR, the penalty and solver parameters were set to L2 and ‘newton-cg’, respectively. For Adaboost, the maximum number of estimators was set to 1200. The rest parameters were set to their default values. All classifiers were trained using Python Scikit-learn package [107].

#### 3.2.4 Performance evaluation

For evaluation, a five-fold cross-validation technique was used. Cross-validation is a common validation process used to evaluate machine learning models and increase the reliability of the results in the case of limited data [108]. The data in this approach is divided into five groups. For each group, the data of that group is held out as a test set where all the remaining groups are used as training sets. The splits in each fold were stratified to preserve the samples’ distribution in each emotion. The resultant confusion matrix was formed by adding up the confusion matrices from all five folds. The overall performance of the classifier is determined by the average performance for all test sets. For each classifier, four performance metrics were calculated which are accuracy, unweighted average recall (UAR), unweighted average precision (UAP), and unweighted average F-score (UAF).

#### 3.2.5 Results and discussion of the objective evaluation

**Fig 9:**
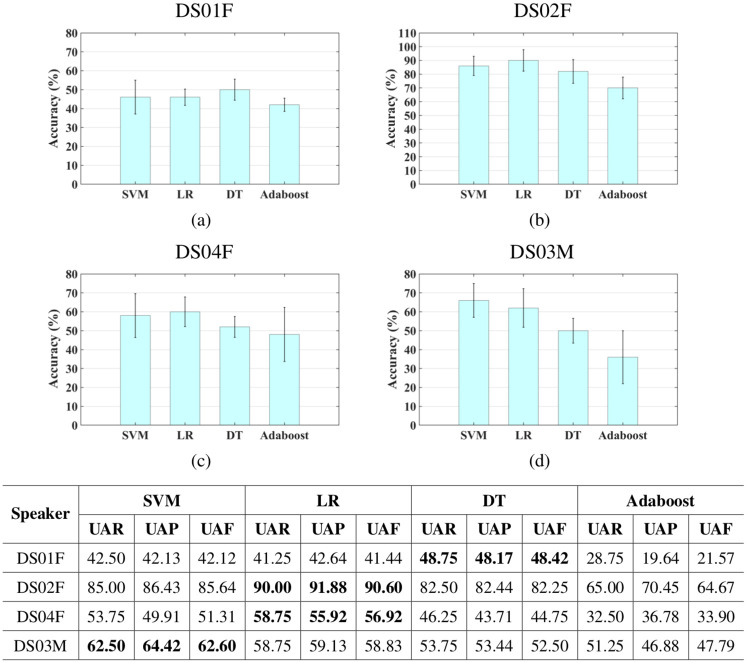
Speaker-dependent categorical classification results using 4 emotions with error bars show the 95% confidence interval.

The experimental results for the categorical classification approach using 7 emotions and 4 emotions are presented in Table 9 and Fig 9, respectively.

**Table 9:** Speaker-dependent categorical classification results using 7 emotions.

Speaker	SVM				LR			
	Accuracy	UAR	UAP	UAF	Accuracy	UAR	UAP	UAF
DS01F	**28.75**	**27.14**	27.84	27.16	27.50	25.00	26.82	25.32
DS02F	**67.50**	**64.29**	62.55	62.57	63.75	60.00	59.77	59.65
DS04F	28.75	23.57	19.59	21.37	**37.50**	**32.86**	31.22	31.97
DS03M	**47.50**	**43.57**	44.07	42.83	43.57	41.43	40.77	40.58

The performance of all classifiers using the full set of emotions and the reduced set are above chance performance, even for speaker DS01F who has severe dysarthria and low speech intelligibility. In fact, the results of classifying 7 emotions generally outperforms the human performance reported in Table 5 on all speakers except on speaker DS04F. The performance of the classifiers improved for all speakers when the number of emotions were reduced as would be expected. In all of the experiments, highest classification performance is achieved on speaker DS02F. An accuracy of 67.50% using SVM and 90% using LR is achieved using 7 and 4 emotions, respectively. This could be due to this speaker having mild dysarthria. The performance of the different classifiers are comparable. In fact, by looking into the 95% confidence interval of the classifiers accuracy in Fig 9, it could be inferred that the differences between the classifiers accuracy, for each speaker, are most generally not statistically significant.

**Fig 10:**
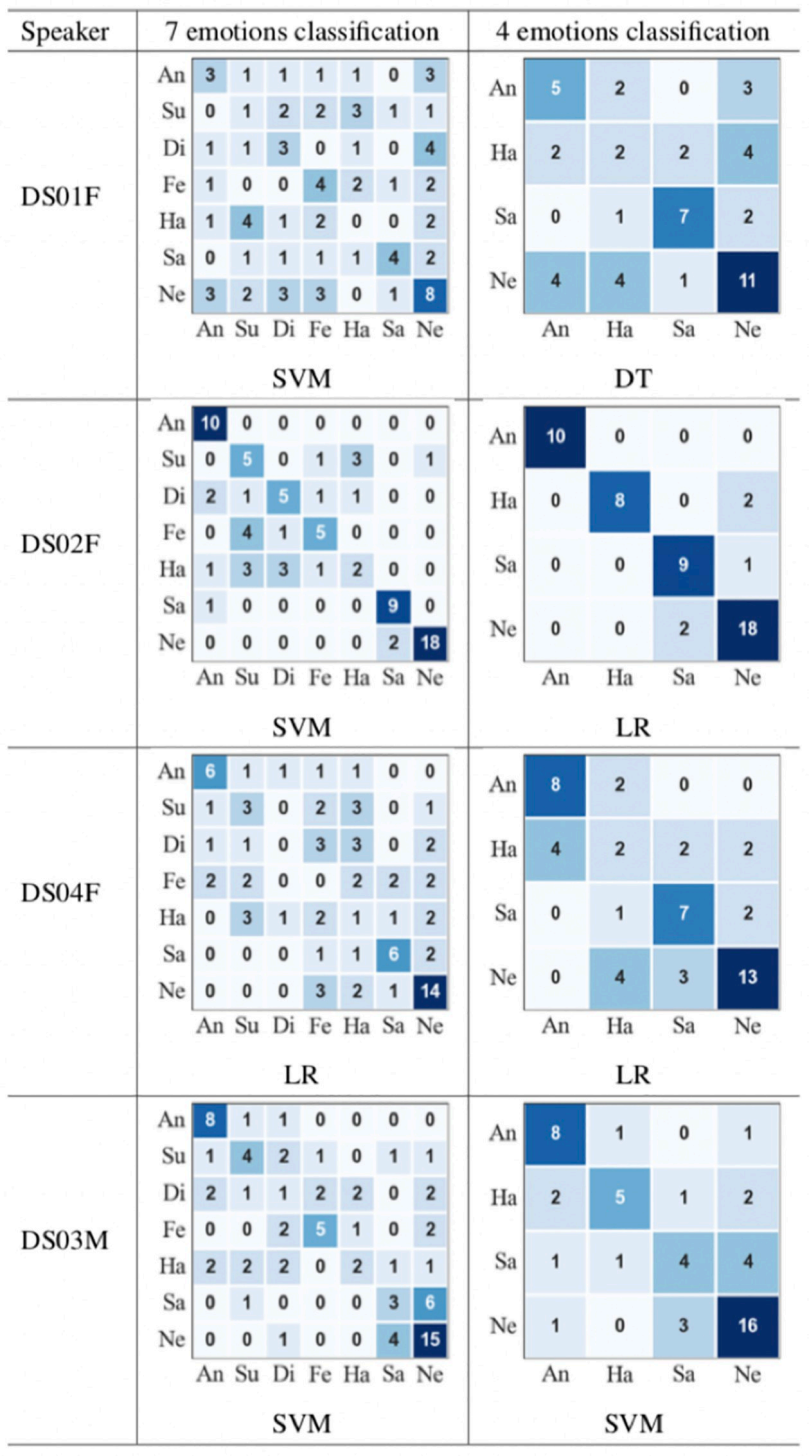
Confusion matrices of the categorical classification using 7 and 4 emotions. (rows = actual emotions and columns = classified emotions, An = angry, Su = surprise, Di = disgust, Fe = fear, Ha = happy, Sa = sad, and Ne = neutral).

In addition to general performance metrics, confusion matrices help in giving more information about the classification performance. Fig 10 presents the confusion matrices for the classification approach using 7 and 4 emotions for all speakers. For each speaker, the confusion matrix of the best classifier is presented where the rows present the actual emotions and the columns present the classified emotions. From Fig 10, it is observed that for all speakers, when classifying the full set of emotions and the reduced set, i) ‘anger’ is never confused with ‘sad’ and ‘sad’ is rarely confused with ‘anger’, ii) ‘sad’ is mostly confused with ‘neutral’, iii) for all speakers, except speaker DS01F, ‘anger’ and ‘neutral’ are mostly considered as non-confusing pairs, and iv) ‘anger’ appears to be the easiest emotion to classify while ‘happy’ appears to be the most difficult emotion to classify. Similar results were observed on typical speech in [109, 110]. It is also observed that ‘happy’ and ’surprise’ are mostly confused with each other. The biggest improvement is achieved for classifying ‘happy’ for all speakers when reducing the classification problem to 4 emotions.

Developing an SER on *typical* speech is not the focus of this paper, however, it would be useful to have a baseline results on the typical speech part of DEED. Setting a baseline is important to be able to compare it to the results obtained from the dysarthric SER. Also, it will give an insight into the level of difficulty of the classification problem. Therefore, using the same settings in terms of the feature set and classification tasks, a SER on the typical speech part of the DEED, was developed. The performance of the speaker-dependent categorical classification approach using two classifiers, OVR-SVM and LR were tested. The average accuracy of classifying 7 emotions is 50.45% (Minimum = 38.75%, Maximum = 66.25%) and 55.61% (Minimum = 32.50%, Maximum = 73.50%) using OVR-SVM and LR, respectively. These classification performances are comparable to what is achieved on another emotional typical database. These results show that this is a difficult task even when staying within the typical domain.

## 4. Conclusion

This paper described the development and design of DEED. DEED has a number of important features that we believe that scientists and clinicians will be interested in. It is a parallel database of typical and dysarthric emotional speech which allows direct comparison and analysis to be made between those two types of speech. To the best of our knowledge, there are no dysarthric emotional speech databases nor databases with this parallelism feature of typical and dysarthric emotional speech. It is a multimodal database which allows the integration and analysis of two modalities, audio and video. All design methodologies have been selected carefully to best serve the purpose of this database. The database is freely available for the research community.

In order to determine the adequacy and quality of the DEED recordings, a subjective evaluation was performed. The initial plan was to recruit normal hearing participants from three different groups: participants who are familiar with dysarthric speech and familiar with a speaker/speakers with dysarthria in DEED, participants who are familiar with dysarthric speech but are not familiar with any of the speakers with dysarthria in DEED, and participants who are not familiar with dysarthric speech at all. The aim was to compare the effect of familiarity with the speech and speakers on the recognition of emotions. However, due to the situation of COVID-19 and the lockdown imposed, it was difficult to recruit enough participants from each group and analyse their results separately. A detailed analysis will be done in the future including participants from each group and applying statistical measures to check the significance, if any, of the participants familiarity level on the recognition performance.

Nevertheless, the conducted evaluation indicated that speakers with dysarthria in DEED were able to communicate different emotions. The overall recognition performance showed that participants in this study were able to recognise emotions spoken by speakers with dysarthria even for speaker DS01F, who has severe dysarthria and highly unintelligible speech. These results demonstrated that this database will be a useful resource for understanding emotion communication by people with dysarthria. They also validate the use of the database in acoustic and modelling studies [36].

After that, a dysarthric SER model was implemented and the results on four speakers with dysarthria were presented and discussed. Given the nature of the dysarthric speech and its phonological and prosodic dimensional limitations, the experiments were conducted to investigate i) the feasibility of automatically recognizing emotions from dysarthric speech, and ii) what emotions in the dysarthric speech are found to be close to each other (confusing) in the chosen feature space. It was demonstrated that dysarthric speech emotion recognition could be possible. In fact, the results of recognising 7 and 4 emotions using categorical classification approach were very encouraging including the results of speaker DS01F who has severe dysarthria and highly unintelligible speech. All results were above chance performance which confirms the initial findings from the performed subjective evaluation that people with dysarthria may have control to perform systematic changes in their speech to communicate emotions. It was observed in all of the experiments that the performance of the classifiers increased when the number of emotions decreased. This is expected as the classification problem gets simpler in terms of the number of classes that the data will be classified into. Thus, generally speaking, the performance using 4 emotions is better than using 7 emotions.

Most deep learning techniques require a large amount of data to be able to perform well. However, collecting dysarthric emotional speech is more challenging than typical speech data. Therefore, given the encouraging results obtained from the speaker-dependent dysarthric SER models presented in this paper, it would be interesting to investigate whether a dysarthric SER model would benefit from being trained on typical speech data. In other words, investigating whether people with dysarthria share some similarities with typical speakers while expressing emotions and to what extent models trained on typical speech data can accurately classify emotions in dysarthric speech. An initial investigation has already been performed and the encouraging results can be found in [37]. The relatively good results when testing the dysarthric speakers on models trained on typical speech indicates that a good level of typical-like emotion specific information is being successfully expressed.

In conclusion, the work presented in this paper showed that people with different etiology and severity of dysarthria can communicate different emotions through their speech. A significant and important finding is that these emotions can be picked up using automatic processing. Also, the results obtained from the different experiments presented in this paper demonstrated that this database will be a useful resource in the field of dysarthric emotion classification research field. The research presented in this paper could be seen as the starting point to a more deep focused investigation of the research problem. Several future directions for this work are suggested below.

- Increasing the size of the recorded database, DEED, by recruiting and recording more speakers with dysarthria and more aged-matched typical speakers. Having more data will allow the use of state of the art SER methods which will enhance the emotion classification performance.
- Comparing the effect of familiarity with the dysarthric speech and speakers on the classification of emotions. This would imply augmenting the subjective evaluation done by recruiting more participants from three different groups as explained earlier.
- Applying different data augmentation techniques on DEED and exploring the classification performance of different deep learning models.
- Exploring the visual part of DEED. Investigating the ability of people with dysarthria perceptually and objectively to communicate emotions through facial expressions and gestures. If they were able, then investigate the effect of combining audio and video emotional cues to the classification performance.

## Supporting information

S1 Table List of DEED sentences. (PDF)

S1 Fig Subjective evaluation—(a) instructions screen and (b) training screen. (TIF)

S2 Fig Subjective evaluation—evaluation screen (a) before playing the recordings and (b) after playing the recordings. (TIF)
