# Supplementary material for: The Dysarthric Expressed Emotional Database (DEED): An audio-visual database in British English
Source: PLoS One. 2023 Aug 7;18(8):e0287971. doi: 10.1371/journal.pone.0287971 (PMC10406321; doi:10.1371/journal.pone.0287971)
Supplement: S1 Table — (PDF) [file pone.0287971.s001.pdf]

List of Dysarthric Expressed Emotion Database(DEED) sentences for Anger, Disgust, Fear, Happiness, Sadness, Surprise and Neutral emotions

Color Code	
	Common Sentences
	Emotion Specific
	Generic Sentences

Emotion	DEED Sentence Number	DEED Sentence
Neutral	01	She had your dark suit in greasy wash water all year.
	02	Don't ask me to carry an oily rag like that.
	03	Will you tell me why?
	04	The best way to learn is to solve extra problems.
	05	Calcium makes bones and teeth strong.
	06	Greg buys fresh milk each weekday morning.
	07	He always seemed to have money in his pocket.
	08	No return address whatsoever.
	09	Who authorized the unlimited expense account?
	10	Destroy every file related to my audits.
	41	Please take this dirty table cloth to the cleaners for me.
	42	The small boy put the worm on the hook.
	43	Call an ambulance for medical assistance.
	44	Tornadoes often destroy acres of farm land.
	45	The carpet cleaners shampooed our oriental rug.
	46	His shoulder felt as if it were broken.
	47	The prospect of cutting back spending is an unpleasant one for any governor.
	48	The diagnosis was discouraging; however, he was not overly worried.
	49	Those musicians harmonize marvelously.
	50	The eastern coast is a place for pure pleasure and excitement.
Anger	11	She had your dark suit in greasy wash water all year.
	12	Don't ask me to carry an oily rag like that.
	13	Will you tell me why?
	14	Who authorized the unlimited expense account?
	15	Destroy every file related to my audits.
	51	Cory and Trish played tag with beach balls for hours.
	52	He will allow a rare lie.
	53	Withdraw all phony accusations at once.
	54	Right now may not be the best time for business mergers.
	55	A few years later the dome fell in.
Disgust	16	She had your dark suit in greasy wash water all year.
	17	Don't ask me to carry an oily rag like that.
	18	Will you tell me why?
	19	Please take this dirty table cloth to the cleaners for me.
	20	The small boy put the worm on the hook.
	56	Basketball can be an entertaining sport.
	57	How good is your endurance?
	58	Barb burned paper and leaves in a big bonfire.
	59	If the farm is rented, the rent must be paid.
	60	Laboratory astrophysics.
Fear	21	She had your dark suit in greasy wash water all year.
	22	Don't ask me to carry an oily rag like that.
	23	Will you tell me why?
	24	Call an ambulance for medical assistance.
	25	Tornadoes often destroy acres of farm land.
	61	Straw hats are out of fashion this year.
	62	That diagram makes sense only after much study.
	63	Special task forces rescue hostages from kidnappers.
	64	Will Robin wear a yellow lily?
	65	The pulsing glow of a cigarette.
Happines	26	She had your dark suit in greasy wash water all year.
	27	Don't ask me to carry an oily rag like that.
	28	Will you tell me why?
	29	Those musicians harmonize marvelously.
	30	The eastern coast is a place for pure pleasure and excitement.
	66	Project development was proceeding too slowly.
	67	The oasis was a mirage.
	68	Are your grades higher or lower than Nancy's?
	69	Serve the coleslaw after I add the oil.
	70	He would not carry a brief case.
Sadness	31	She had your dark suit in greasy wash water all year.
	32	Don't ask me to carry an oily rag like that.
	33	Will you tell me why?
	34	The prospect of cutting back spending is an unpleasant one for any governor.
	35	The diagnosis was discouraging; however, he was not overly worried.
	71	Before Thursday's exam, review every formula.
	72	They enjoy it when I audition.
	73	John cleans shellfish for a living.
	74	He stole a dime from a beggar.
	75	American newspaper reviewers like to call his plays nihilistic.
Surprise	36	She had your dark suit in greasy wash water all year.
	37	Don't ask me to carry an oily rag like that.
	38	Will you tell me why?
	39	The carpet cleaners shampooed our oriental rug.
	40	His shoulder felt as if it were broken.
	76	The viewpoint overlooked the ocean.
	77	I'd ride the subway, but I haven't enough change.
	78	The clumsy customer spilled some expensive perfume.
	79	Grandmother outgrew her upbringing in petticoats.
	80	Salvation reconsidered.
