# Supplementary figures and images for: The Dysarthric Expressed Emotional Database (DEED): An audio-visual database in British English
Source: PLoS One. 2023 Aug 7;18(8):e0287971. doi: 10.1371/journal.pone.0287971 (PMC10406321; doi:10.1371/journal.pone.0287971)

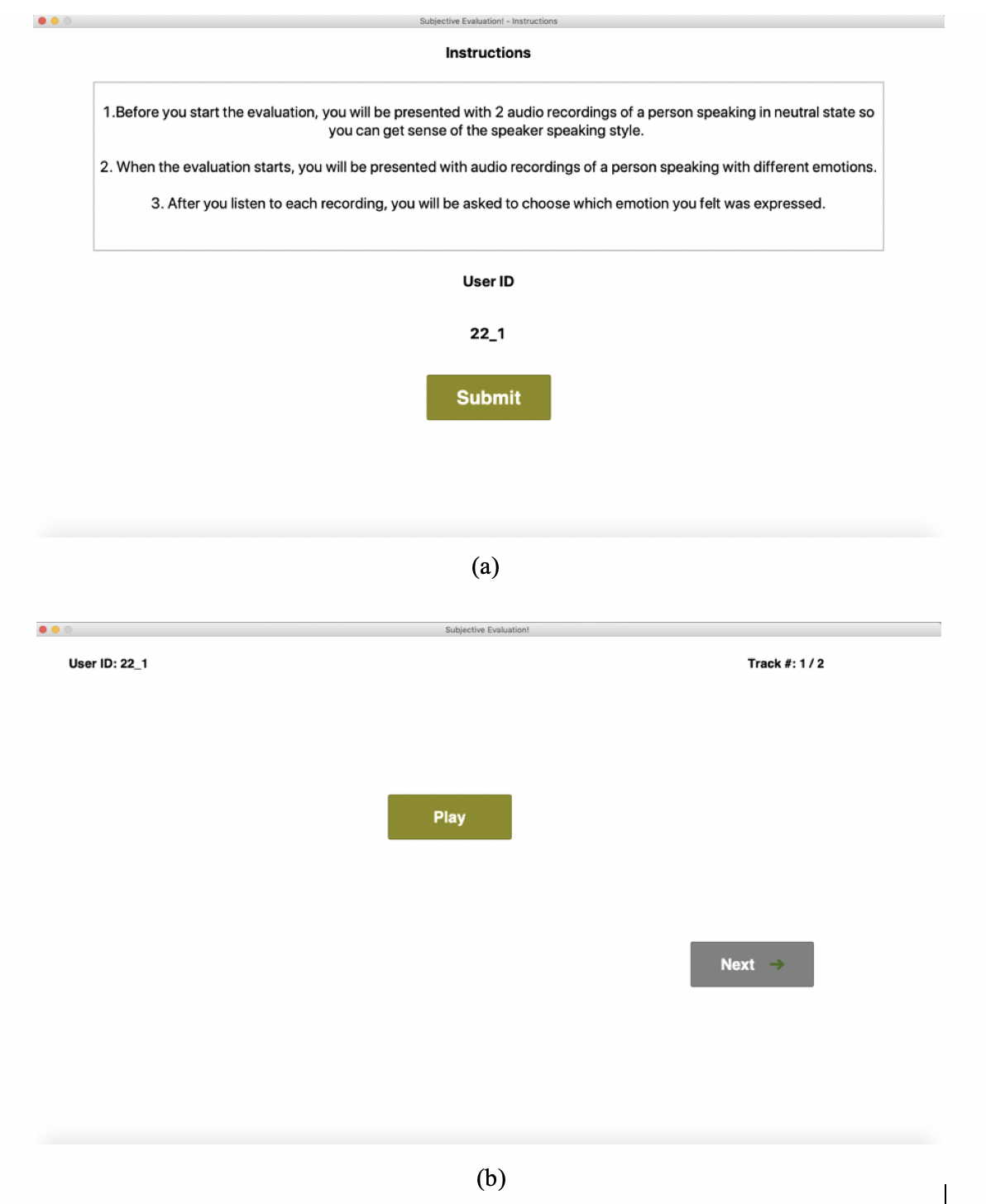

Supplement: S1 Fig — (TIF) [file pone.0287971.s002.tif]

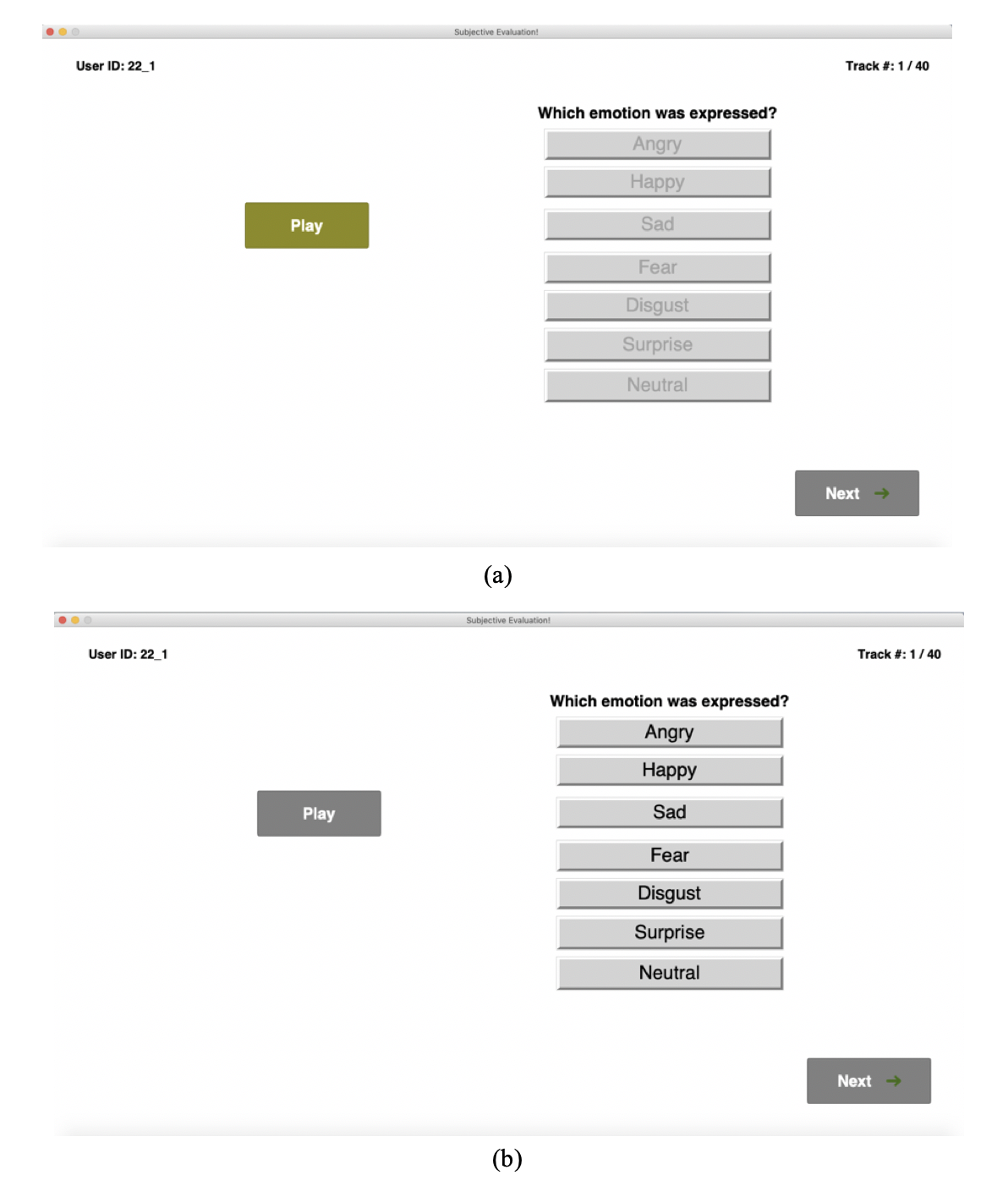

Supplement: S2 Fig — (TIF) [file pone.0287971.s003.tif]
